# Tetramethylpyrazine in Chinese baijiu: Presence, analysis, formation, and regulation

**DOI:** 10.3389/fnut.2022.1004435

**Published:** 2022-09-20

**Authors:** Xiaoshan Shi, Shumiao Zhao, Shenxi Chen, Xinglin Han, Qiang Yang, Long Zhang, Xian Xia, Junming Tu, Yuanliang Hu

**Affiliations:** ^1^Key Laboratory of Edible Wild Plants Conservation and Utilization, Hubei Normal University, Huangshi, China; ^2^Hubei Engineering Research Center of Characteristic Wild Vegetable Breeding and Comprehensive Utilization Technology, Huangshi, China; ^3^State Key Laboratory of Agricultural Microbiology, College of Life Science and Technology, Huazhong Agricultural University, Wuhan, China; ^4^Jingpai Co. Ltd., Daye, China; ^5^Beijing Laboratory for Food Quality and Safety, Beijing Technology and Business University, Beijing, China

**Keywords:** Chinese baijiu, health flavor factor, TMP, formation mechanism, regulation strategies

## Abstract

Traditional Chinese fermented baijiu is one of the six major distilled spirits consumed worldwide. It plays an important role in people's daily life and social interactions because of its taste, nutritional value, and various health functions. Tetramethylpyrazine (TMP), also known as ligustrazine, is not only an important compound related to the flavor of Chinese baijiu but also has special pharmacological effects. It gives the baijiu a nutty and baked aroma and provides baijiu with important health benefits. Recently, the nutritional, drinking, and health aspects of baijiu have attracted significant attention. Therefore, the study of TMP in baijiu is an important aspect of baijiu health research. This mini novel review summarizes the formation mechanism of TMP, along with the current research progress, analytical methods used, and regulation strategies associated with TMP in Chinese baijiu in recent years.

## Introduction

**Figure 1:**
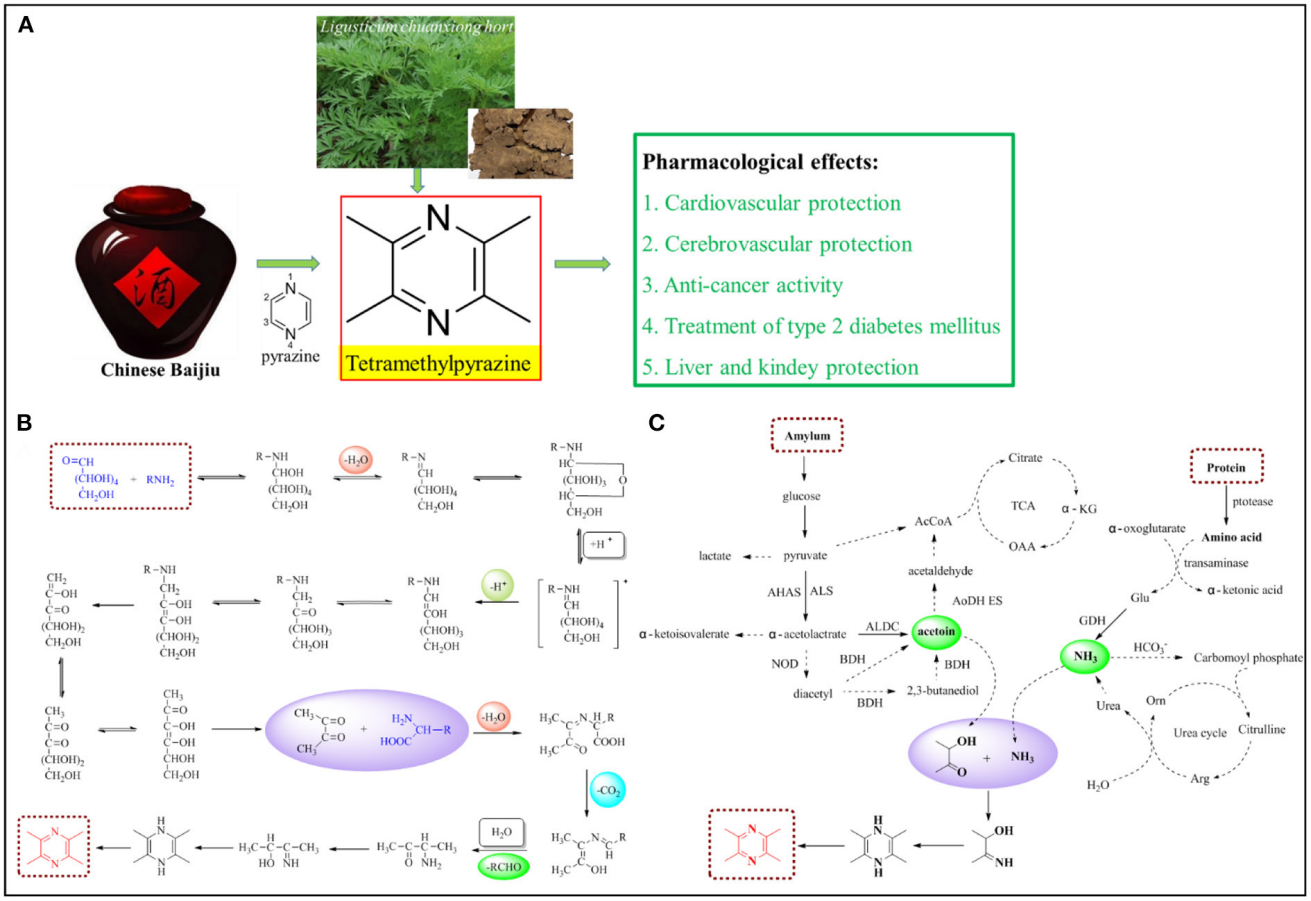
Structure and functions of TMP **(A)**; the Maillard reaction **(B)** and microbial synthesis **(C)** to produce TMP.

As China's national liquor, baijiu is still favored by modern people even after thousands of years of inheritance, which not only highlights the allure of Chinese baijiu but also its value as a health product (1, 2). The unique brewing process of Chinese baijiu produces many beneficial trace ingredients besides alcohol, including phenols (3), terpenes (4), pyrazines (5), amino acids (6), and polypeptides (7). Among them, pyrazines are six-membered heterocyclic compounds with two nitrogen atoms at positions 1 and 4 (Figure 1A). Tetramethylpyrazine (TMP), with methyl groups attached to all the carbon atoms of the pyrazine ring (Figure 1A), is the most abundant pyrazine compound in baijiu (8, 9). It is widely found in raw foods, processed foods, and alcoholic beverages (10). It has a pleasant aroma of roasted peanuts, hazelnut, and cocoa, and is considered an important aromatic compound (11). In addition to being a flavor additive in food, TMP has great nutritional value. Therefore, TMP not only contributes significantly to the flavor of Chinese baijiu but also endows it with health functions.

TMP, also known as ligustrazine, is an active pharmaceutical ingredient that was originally isolated from the traditional Chinese medicine plant *Ligusticum chuanxiong*. It can dilate blood vessels, inhibit platelet aggregation, improve microcirculation, increase coronary and cerebral blood flow (12, 13), and is a commonly used antiplatelet drug in clinics (14). It can prevent various diseases, such as cardiovascular and cerebrovascular diseases, ischemic stroke, cancer, and diabetes (15, 16). Therefore, the study of TMP in baijiu is an important field of health research. This review focuses on TMP in baijiu and provides a comprehensive summary of its concentration, aroma contribution, and health effects. This review also discusses the microbial metabolic pathways for the synthesis of TMP. These summaries will provide detailed understanding of baijiu flavors from the perspective of TMP.

## Concentration and health functions of TMP

### Concentration of TMP in baijiu

Chinese baijiu is divided into the following 12 flavor types: sauce-, strong-, rice-, light-, chi-, feng-, sesame-, drug-, sauce-strong-, laobaigan-, te, and fuyu-flavor (6). TMP can be detected in different flavor types of baijiu (5, 17), but the contents differ depending on the raw materials, baijiu-brewing technology, and control conditions used. The contents of TMP in several typical baijius are listed in Table 1 (18–25). The content of TMP in sauce-, sesame-, and rice-flavored baijiu is higher than that in light-flavored baijiu. The high content of TMP is a common characteristic of sauce-flavored baijiu, and it increases with increasing alcohol content.

**Table 1:** Summary of TMP detected in different types of baijiu.

**Flavor types and baijiu brands**	**Concentration of TMP (mg/L)**	**References**
**Sauce flavor type**		
MaoTai	30.782; 53.02;	(18)
	4.40	(19)
	3.88; 4.89; 20.15 (53%vol)	(20)
LangJiu	0.731; 1.27; 0.17	(18, 19, 21)
	1.36 (10 years); 1.72 (53%vol)	(20)
ZhenJiu	2.14 (10 years); 2.45 (15 years)	(20)
JinMao	4.92	(20)
FeiTian MaoTai	6.52	(20)
LaiMao	1.18	(20)
MaoTai YingBinJiu	4.32	(20)
TuMaoJiu	2.26 (8 years)	(20)
JinMaoChen	1.58 (5 years)	(20)
LaiJiu	1.22 (5 years)	(20)
LaiShiGang	1.15	(20)
JinGuiPiaoXiangJiu	1.41	(20)
TanJiu	2.54	(20)
WuLinZhongJiang	1.69	(20)
YunMen	0.43; 1.25	(21)
GuiZhouYingBinJiu	1.38 (8 years)	(20)
DaoYuTai	1.72 (53%vol)	(20)
LaoTuJiu	1.52	(20)
HuTuLaoJiangJiu	2.63	(20)
QiBin (GuoTai)	1.75	(20)
HongSiDu	1.62 (5 years)	(20)
ZhenJiuZhenPin	1.48	(20)
QianGuanWang	1.87	(20)
JiuShenJiu	1.58	(20)
JinShaHuiShaJiu	4.82	(20)
**Strong flavor type**		
WuLiangYe	0.195; 0.50	(18, 19)
ShuangGouDaQu	0.12	(18)
ShangZhuangLaoQu	0.1	(22)
YangHeDaQu	0.023	(18)
**Light flavor type**		
FenJiu	0.075	(18)
**Rice flavor type**		
ZhiJiangBaiJiu	10.36	(23)
**Sesame flavor type**		
MeiLanChun	0.71	(24)
GuoJin	0.4	(24)
ShengLiYuan	0.3	(24)
BanDaoJingJiu	0.46	(24)
XuanJiuZhiXiang	1.26	(25)
**Sauce strong flavor type**		
JingZhi	0.156; 0.16	(18, 24)
BaiYunBian	0.482; 0.75	(18, 19)

### TMP impact on health

*Ligusticum* was first observed in Shennong Ben Cao Jing. In traditional Chinese medicine, it is believed to replenish blood, promote blood circulation, and disperse blood (26). TMP, the main active substance of *Ligusticum chuanxiong*, has attracted the interest of many researchers since it was first isolated in 1957 (15, 16). TMP plays a role in various diseases, of which cardiovascular and cerebrovascular diseases are the most prominent. It has been officially listed in the Chinese Pharmacopeia (2010 expanded and revised edition, First part, Medicinal Herbs and Decoction Pieces) for the treatment of the aforementioned diseases (16). Phosphate and hydrochloride formula injections are prescription drugs that are available in China.

TMP has great potential and necessitates further research. In recent years, Lin et al. (15) reviewed various physiological functions of TMP (Figure 1A), which can be summarized as follows: (1) TMP (5–150 mg/kg) reduces low-density lipoprotein oxidation and inflammation, inhibits platelet activation, alleviates myocardial ischemia/reperfusion injury, and has a positive therapeutic effect on patients with cardiovascular diseases. (2) TMP (10–200 mg/kg) can penetrate the blood–brain barrier and promote angiogenesis, and it plays a role in cerebrovascular diseases and neuroprotection through its anti-apoptotic, anti-inflammatory, and antioxidant pathways. (3) TMP (30–500 mg/kg) can induce cell apoptosis, block angiogenesis, and play an anticancer role. (4) TMP (15–200 mg/kg) can improve the systemic insulin resistance and play an important role in diabetic complications. (5) TMP (20–200 mg/kg) can regulate lipid metabolism, fight lipid peroxidation and tissue fibrosis, and impart protective effects on the liver and kidneys. (6) TMP (50–150 mg/kg) can also improve the progression of neurodegenerative diseases. From Table 1, we can observe that the concentration of TMP in several sauce-flavored baijius is in the above range, which can directly exert its health effect.

At present, the most studies on the role of TMP in cardiovascular diseases have been focused on animal and cell models, with limited clinical trial data. Therefore, large-scale clinical trials are required to evaluate the clinical efficacy of TMP.

## Analysis method of TMP

The pyrazine content, especially TMP, in baijiu is minimal. The content of TMP in baijiu mostly ranges from a few micrograms to tens of milligrams per liter, and its composition determination is susceptible to interference from other volatile substances. Therefore, it is of great significance to study the qualitative and quantitative methods used to determine the TMP content in baijiu, including sample extraction and separation.

The main methods of sample extraction are direct injection, liquid–liquid extraction (LLE), liquid–liquid microextraction (LLME), solid-phase extraction, and solid-phase microextraction (SPME). The direct injection method reduces the loss of the target compound and has a higher quantitative accuracy. However, the interference of other compounds affects the quantitative determination of TMP to a certain extent. LLE allows the removal of water, ethanol, and volatile components that interfere during the analysis and is therefore more beneficial for the determination of TMP. LLE combined with gas chromatography–mass spectrometry (GC–MS) has been used to analyze pyrazines in strong-flavored baijiu, and the TMP content is observed to be above 100.49 *u*g/L (22). However, LLE includes additional steps, such as acidizing liquid extraction, alkali conditioning, re-extraction, and concentration. Therefore, the more complex process may lead to the loss of TMP and thus affect the accuracy of the quantitative analysis. Vortex-assisted LLME requires fewer organic solvents and is easier to operate. Scroll-assisted LLME combined with GC–MS has been used to detect TMP in 67 types of baijiu and found that all samples contained TMP, with the TMP content ranging from 1.5 to 2,434.3 μg/L (27). SPME is a novel, solvent-free sample microextraction technology that integrates sampling, extraction, concentration, and injection. It overcomes the disadvantage of analyte loss in traditional analytical methods, although it has a high cost. Wang et al. (28) detected TMP in sauce- and strong-flavored baijiu using headspace SPME. The two pretreatment methods of SPME and vortex-assisted LLME (VA-LLME) was compared and found that VA-LLME was more suitable for the study of volatile active components in raw wine (29).

The main methods of sample separation and analysis are GC, GC–MS, GC–MS/MS, and liquid chromatography (LC). Traditional GC is based on one-dimensional chromatography, which has insufficient peak capacity. It is difficult to obtain satisfactory analysis results by only improving the column efficiency or selectivity. In comprehensive two-dimensional gas chromatography (GC × GC), two chromatographic columns with different separation mechanisms are connected in a series, which significantly improves the chromatographic peak capacity and has the characteristics of high sensitivity, considerable peak capacity, and high resolution; thus, it has unique advantages in the analysis of a variety of trace aroma components in distilled liquor systems. Using GC × GC-TOFMS, more than 100 functional components was identified, such as TMP, in Jiannanchu baijiu (30). The scan method, commonly used in traditional GC–MS, is also limited by interferences from other pyrazines, esters, and alcohols, which affect the quantification to a certain extent. The selective ion monitoring (SIM) method is more commonly used to detect substances with lower contents because it only monitors the target compound and rejects the interference information of other compounds. The sensitivity of this method is much greater than that of the scan method. Using GC/MS–SIM, the content of TMP in baijiu was determined by direct injection (t_R_ 4.57 min) (19) or LLE (t_R_ 16.30 min) (31), and was much higher in maotai-flavored baijiu than in sesame- and luzhou-flavored baijiu. The triple quadrupole GC–MS has been used to determine the content of TMP (t_R_ 28.246 min) and achieved accurate qualitative and quantitative determination of the target substance through secondary collision, which has the advantages of rapidity, accuracy, good selectivity, and high sensitivity (32).

In view of the existing detection methods for TMP, establishing a simple and accurate method to determine the TMP content is of great significance for the study of TMP in baijiu, the daily analysis and inspection of the distillery, and the promotion of its own brand. Researchers have successively established a method for the determination of TMP using the GC internal standard method (t_R_ 18.207 min) (25, 33), GC external standard method (t_R_ 16.401 min) (33, 34), and LC-MS combined with the external standard method (t_R_ 3.592 min) (33, 35). The recovery and precision of these three methods fulfill the daily analysis requirements of baijiu. Furthermore, LC-MS has been found to be the best method for determining TMP in baijiu (33).

## TMP generation in baijiu

TMP in baijiu can be produced in different degrees during qu-making, stacking fermentation, and distillation, and is delivered into baijiu by distillation. There are currently two ways to produce TMP: the Maillard reaction and microbial synthesis (36). In qu-making and fermentation stages, TMP is mainly generated by microbial metabolism, while in distillation stage, it is mainly generated by Maillard reaction (36, 37).

### TMP generated by the maillard reaction

The Maillard reaction products contain melanoids, reductones, and a series of volatile heterocyclic compounds containing nitrogen or sulfur, with TMP has a higher content and stronger activity. TMP can be produced by condensation of the Maillard reaction intermediate butanedione with amino acids from protein hydrolysis during brewing (Figure 1B) (38). High-temperature qu-making, stacking, and distillation are key elements for the formation of Maillard products (39). Tan et al. found that in distillates containing glucose and glycine, the glucose and glycine contents significantly increased, as well as the contents of the Maillard reaction products, among which TMP increased by 48.96% (40). The amount of TMP was affected by the type and concentration of the Maillard reaction substrates. Heating also favors the generation of TMP, and the production of TMP increases with time (41).

### The role of microorganisms in TMP generation

As early as 1962, Kosuge et al. (42) isolated TMP from the fermented soybean product “natto,” and found for the first time that *Bacillus natto* had the ability to biosynthesize TMP. In addition, many scholars found that TMP could also be produced by fermentation of *Lactococcus lactis* subsp, *Bacillus* sp, *Saccharomyces cerevisiae*, and *Corynebacterium glutamicum* (43, 44).

There are many microorganisms in liquor yeast, fermented grains, and pit mud. These microorganisms, especially some functional microorganisms, play an important role in the quality of baijiu. Studies have found that high-temperature resistant *Bacillus subtilis, Bacillus licheniformis, Bacillus giant, Bacillus pumilus, Bacillus amylolitica, Bacillus methylotrophic, Bacillus proteolysaccharide*, and *Bacillus brevis* could all metabolize and produce TMP under jiuqu culture conditions (44–50). In 2018, it was first found that the high-temperature actinomycete strain *Laceyella Sacchari* also metabolizes and produces TMP under solid-state fermentation conditions (51). Through the screening of *Bacillus subtilis* and analysis of the metabolic mechanism of the functional strain with a high yield of TMP precursor in Chinese baijiu, the microbial production mechanism of TMP in Chinese baijiu was determined. The functional strain degrades sugar to produce pyruvate, and the two molecules of pyruvate condense to form α-acetolactrate, followed by the decarboxylation of α-acetolactrate to yield acetoin. Acetoin, in the fermentation system, and ammonia, mainly converted from amino acids, undergo non-enzymatic reactions to form TMP (Figure 1C) (36, 52).

The microbial sources of TMP in Chinese baijiu demonstrate the important role of liquor functional microorganisms in liquor brewing and the significance of this field of research. Recently, many famous enterprises in China have conducted research on TMP production by microorganisms to improve the content of TMP in baijiu (53).

## Regulation strategies of TMP

TMP is a recently discovered health factor in baijiu. Increasing the TMP content is of great significance for the flavor and quality of baijiu. Therefore, improving the TMP content has become a notable research topic in the field of liquor fermentation. The TMP content in different flavored baijius differs depending on the raw materials and technology used for liquor preparation. Thus, the technological process and microbial properties can be improved by increasing the TMP content in baijiu.

### Raw material

The type and concentration of raw materials are important factors affecting the reaction products. The TMP content was significantly higher in the baijiu brand with sorghum as the raw material and wheat as the main ingredient (27). Natural and intrinsic factors cause the surface and internal tissues of raw materials from different origins to form unique microbial communities, which directly or indirectly affect the quality and flavor of baijiu (54). Therefore, in liquor brewing, the selection of raw materials, increasing the concentration of raw materials, and fermentation strength can increase the TMP content.

### Enhanced fermentation of functional microorganisms

Based on the characteristics of the microbial synthesis of TMP, the addition of *Rhizopus* qu with a strong saccharification ability is conducive to the reduction of sugars. In the early fermentation process, yeast produces a variety of enzymes that decompose the raw materials and promote the generation of precursor substances. After the fermentation temperature rises, the yeast ceases to exist and acts as a nitrogen source to promote the growth of heat-resistant bacteria, such as *Bacillus*. Therefore, the TMP content could be effectively increased by inoculating yeast and rhizopus, followed by inoculating functional bacteria with a high TMP yield (55). Using four strains of high-yielding TMP *Bacillus strains* during the brewing of raw wine significantly increased the TMP content in the original wine to more than 10 mg/L (53). The TMP content in sesame-flavored baijiu increased from 0.4 to 1.8 mg/L by adding *Bacillus* sp. Wang et al. (56) added *Bacillus licheniformis* to the fermented grains in the middle layer of the pit. When the added amount of *Bacillus licheniformis* was 5%, the TMP content of the fermented grains was 6.81 μg/g, which was 3.03 times more than that of the control group, and the sensory quality of the fermented grains was significantly better than that of the control group.

### Accumulation temperature and time

High-temperature deposition provides a variety of complex microorganisms (yeasts, bacteria, and molds) and enzymes for liquor production (47). The higher the temperature and time, the faster the Maillard reaction, and the higher the TMP yield (39). The massive proliferation of yeasts greatly increases the protein content, which indicates that the content of various amino acids is high, providing a material foundation for the formation of TMP. In addition to enriching wild yeast in the air, Italian, ground, or Candida yeast can also be made into bran qu and added to the stacking fermentation process to increase the TMP content (39). The stacking temperature should generally be maintained at 45–50°C to achieve the optimal enzymolysis temperature for various biological enzymes for thermal degradation and non-enzymatic chemical reactions to occur (39). The direct entry of bacteria into the cell increased the TMP content by 4%, whereas the accumulation entry increased the content by 12% (57).

### Optimization of the fermentation process

A high concentration of (NH_4_)_2_HPO_4_ is conducive to the production of TMP, a weakly acidic environment is beneficial for the accumulation of the precursor acetoin, and a neutral environment is favorable for the generation of TMP (56, 58, 59). Therefore, a two-stage pH-control strategy was developed to improve the yield of TMP by maintaining a weakly acidic fermentation broth during the early stage of its cultivation to ensure the proliferation of the cells and accumulation of the precursor acetoin, followed by adjusting the fermentation broth to neutral conditions in the later stage of cultivation to promote the transformation of acetoin to generate TMP. TMP can also be improved by a multi-stage stirring speed coupled with a temperature-controlled fermentation strategy or a glucose and ammonia supplementation strategy (56).

The TMP content in maotai-flavored baijiu could be increased by 160.71, 85.75, and 202.75% by means of strain enhancement, process optimization, and a combination of the two methods, respectively (60). The combination of these methods can further increase the TMP content in baijiu. However, the current research in this area is not sufficient. Therefore, further research is required to determine the optimal raw materials, combination ratio of functional bacteria, and process conditions.

## Discussion

Baijiu has been a valued representation of the wisdom of the Chinese nation for thousands of years. Excavating the bioactive ingredients (healthy functional factors) in Chinese baijiu, scientifically guiding consumers to drink healthily, and promoting the improvement of the liquor industry have been of great interest to scholars at China and abroad. Chinese baijiu, which is rich in health factors and aromas, elegant in taste, and brings health and happiness to individuals, is likely to bring forth sustainable and health-related developments.

## Author contributions

All authors listed have made a substantial, direct, and intellectual contribution to the work and approved it for publication.

## Funding

This work was financially supported by the Hubei Provincial Key Research and Development Program (2020BBA050), the Innovation team Project of Hubei Education Department (T2022010), and the Hubei Key Laboratory of Edible Wild Plants Conservation and Utilization (EWPL202208).

## Conflict of interest

SC, QY, LZ, and YH were employed by Jingpai Co. Ltd. The remaining authors declare that the research was conducted in the absence of any commercial or financial relationships that could be construed as a potential conflict of interest.

## Publisher's note

All claims expressed in this article are solely those of the authors and do not necessarily represent those of their affiliated organizations, or those of the publisher, the editors and the reviewers. Any product that may be evaluated in this article, or claim that may be made by its manufacturer, is not guaranteed or endorsed by the publisher.
